# Assessment of Patient-Reported Outcome Measures Following Pelvi-Acetabular Fractures in Patients Admitted to a Tertiary Care Hospital

**DOI:** 10.7759/cureus.91941

**Published:** 2025-09-09

**Authors:** Sandeep B Rathod, Kshitij Malpani, Rehan Ul-Haq, Aditya N Aggarwal

**Affiliations:** 1 Orthopaedics, University College of Medical Sciences & Guru Teg Bahadur Hospital, New Delhi, IND; 2 Orthopaedics, Rama Medical College, Hospital & Research Centre, Kanpur, IND; 3 Orthopaedics, All India Institute of Medical Sciences, Bhopal, IND; 4 Orthopaedics, University College of Medical Sciences & Guru Teg Bahadur Hospital, Delhi, IND

**Keywords:** patient-reported outcome measures, pelvic acetabulum fractures, pelvic fractures, quality of life, treatment outcome

## Abstract

Background

Pelvi-acetabular fractures can have a profound impact on patient well-being and functional outcomes. Understanding patient-reported outcomes is crucial for assessing the long-term effects of treatment and improving clinical practices. The present study aimed to evaluate patient-reported outcome measures (PROMs) in individuals treated for pelvi-acetabular fractures.

Methods

This study was conducted at a tertiary care hospital and included patients treated for pelvi-acetabular fractures. Data were collected from patients with a minimum follow-up of six months. Both generic PROMs (Short Form-36 (SF-36), Short Musculoskeletal Function Assessment (SMFA), Oxford Hip Score, and Majeed Pelvic Outcome Score) and functional outcome measures (Harris Hip Score (HHS) and Parker Palmer Mobility Score) were recorded. Radiological assessment was performed using Matta grading. Agreement between the SF-36 and HHS was also evaluated.

Results

The mean SF-36 physical functioning component score was 92.28. The SMFA showed a mean score of 7.94. HHS results were good in 53.2% of cases and excellent in 38.3% of cases. Overall, the findings revealed favorable outcomes in terms of general health and functional status after treatment. There was significant agreement between the generic PROM (SF-36) and the disease-specific functional outcome measure (HHS), suggesting overall improvement in health and patient satisfaction.

Conclusion

These findings underscore the importance of comprehensively assessing patient-reported outcomes to capture the full impact of healthcare interventions. The insights from this study may help guide treatment decisions and improve patient satisfaction, particularly in regions with a high incidence of pelvi-acetabular fractures. Further research with larger sample sizes and longer follow-up periods is warranted to refine treatment strategies and enhance the quality of care for individuals with pelvi-acetabular injuries.

## Introduction

The impact of pelvi-acetabular fractures on healthcare outcomes is substantial. Among the various causes of mortality, pelvic fractures stand as the third most common cause of death resulting from road traffic accidents. Disability rates are also high in patients following pelvi-acetabular trauma. The outcome following pelvi-acetabular trauma in patients has been assessed using various functional scores, such as the Harris Hip Score (HHS), Parker-Palmer mobility score, etc., and using radiological Matta grading [1].

Patient-reported outcome measures (PROMs) are validated healthcare questionnaires that patients complete following medical treatment. By focusing on patients' self-reported experiences, PROMs provide a unique perspective that complements traditional clinical assessments. These instruments have emerged as a crucial tool for evaluating the impact of healthcare interventions. They offer a unique perspective, enabling healthcare professionals to understand the patient experience beyond clinical measurements [2].

With this backdrop, the current study seeks to assess the application of PROMs following pelvi-acetabular fractures, given the paucity of literature in this area. The study specifically evaluates quality of life using the Short Form-36 (SF-36), a validated PROM, and complements this with functional outcome assessment using the HHS. Previous studies in this field have focused mainly on radiological and clinician-assessed functional outcomes, with limited exploration of patient-reported quality of life [3]. By examining the patient's perspective and integrating PROMs into the evaluation process, the current research aims to provide insights that can enhance the treatment approach, improve patient satisfaction, and contribute to evidence-based care.

## Materials and methods

This study was conducted at the University College of Medical Sciences and GTB Hospital, Delhi, a tertiary care teaching hospital in India, using a prospective observational design. Data were collected between October 2018 and April 2020.

As this was designed as a prospective observational study in a tertiary care trauma setting, no formal sample size calculation was performed. All eligible consecutive patients presenting during the study period were included to capture real-world data on patient-reported outcomes following pelvi-acetabular fractures. While this pragmatic approach ensured complete enrollment without selection bias, it is acknowledged as a limitation that the relatively small sample size may reduce the statistical power and generalizability of the findings.

Approval from the Institutional Ethics Committee was obtained prior to the commencement of the study.

Written informed consent was obtained from all patients or their caregivers. Patient details were recorded on a predesigned proforma, which included demographic information, date and time of injury, place of occurrence, mechanism of injury, mode of transport to the hospital, associated injuries, treatment received before hospital admission, diagnosis, management provided, treatment of associated injuries, and the patient’s condition at discharge.

The exclusion criteria for the study included patients who presented to the hospital more than 72 hours after sustaining the injury, those with pathological fractures, cases in which a complete radiological workup could not be performed, and pregnant women in whom CT scans were contraindicated.

On admission, patients were initially resuscitated according to the Advanced Trauma Life Support (ATLS) protocol. A thorough clinical examination, including a secondary survey, was performed to rule out missed injuries. Appropriate investigations, including radiographs and CT scans, were conducted to document and classify the fractures.

Acetabular fractures were classified according to the Judet and Letournel system, which distinguishes elementary fracture types such as anterior column, posterior wall, posterior column, and transverse patterns, as well as associated types including T-type, anterior column with posterior hemitransverse, both column fractures, and other unclassified variants [4]. Pelvic fractures were classified using the Tile classification, which categorizes injuries into Type A (stable fractures, such as A1 and A2), Type B (rotationally unstable but vertically stable fractures, such as B2), and Type C (both rotationally and vertically unstable fractures, such as C1 and C3) [5]. In addition, the Young and Burgess classification was applied, which is based on the mechanism of injury and includes lateral compression (e.g., rami fractures, LC1, LC2), anteroposterior compression (APC2, APC3), vertical shear, and combined mechanism injuries [6]. Supportive treatment was provided as clinically indicated.

Management of pelvic and acetabular fractures was either operative or non-operative, depending on the fracture pattern and the decision of the surgical team. Associated injuries were treated as appropriate. Rehabilitation was initiated once the patient’s medical condition allowed, beginning with hip and knee active range-of-motion exercises and individualized weight-bearing restrictions, followed by gradual progression to full weight-bearing with suitable walking aids.

After discharge, patients were followed up regularly in the outpatient department. At each visit, the surgical site and local skin condition were examined, compliance with the rehabilitation protocol was assessed, and recovery was evaluated. Suitable guidance was provided to optimize functional outcomes.

At the six-month follow-up after the injury, patient outcomes were systematically assessed using a predesigned proforma. To ensure clarity regarding outcome measures, we distinguished between PROMs and clinician-administered functional scores. The validated PROM employed in this study was the Short Form-36 (SF-36) questionnaire [7], which evaluates general health status across multiple domains and is widely recognized as a standard PROM. Functional recovery was additionally assessed using the HHS [8]. Although frequently used in orthopedic outcome research, the HHS includes both patient-reported elements (such as pain and daily activities) and clinician-assessed components (such as range of motion and deformity). Therefore, in this study, the SF-36 is reported as the primary PROM, while the HHS is presented as a functional outcome measure.

Radiological assessment was performed with standard radiographs, and the quality of fracture reduction was graded according to Matta’s criteria [9]. Because the primary aim was to evaluate patient perspectives, the agreement between the validated PROM (SF-36) and the functional measure (HHS) was specifically analyzed, with the acknowledgement that the latter is not a pure PROM.

Data were analyzed using IBM SPSS Statistics for Windows, Version 26 (Released 2019; IBM Corp., Armonk, New York, United States). Descriptive statistics were used to summarize the data and expressed as mean with standard deviation (SD), median with interquartile range (IQR), range, and proportions as appropriate. The correlation between patient-reported outcome measures and functional outcome scores was assessed using Spearman’s rank correlation coefficient. A p-value of <0.05 was considered statistically significant.

## Results

The study included a total of 47 patients, with a mean age of 35.55 years (±13.58), ranging from 18 to 65 years. The largest subgroup comprised 15 patients (31.9%) in the 21-30-year age group, followed by 11 patients (23.4%) in the 41-50-year age group. A total of eight patients (17.0%) were between 18 and 20 years of age, while six patients (12.8%) were in the 31-40-year age group. Patients above 50 years accounted for seven cases (14.9%). The detailed age distribution is presented in Table 1.

**Table 1:** Distribution of patients by age

Age Group	Number of Cases (%)
18- 20 years	8 (17.0)
21 – 30 years	15 (31.9)
31 – 40 years	6 (12.8)
41 – 50 years	11 (23.4)
Above 50 years	7 (14.9)

With respect to sex distribution, male patients were more frequently affected than female patients. Out of 47 cases, 30 patients (63.8%) were male, while 17 patients (36.2%) were female, demonstrating a clear male predominance. This pattern may be explained by the higher exposure of men to road traffic accidents, occupational injuries, and outdoor physical activity. The sex distribution of patients is shown in Table 2.

**Table 2:** Distribution of patients by gender

Gender	Number of Cases (%)
Male	30 (63.8)
Female	17 (36.2)

Among the 47 patients, 22 patients (46.8%) had isolated acetabular fractures, 21 patients (44.7%) had isolated pelvic fractures, and four patients (8.5%) sustained combined pelvis and acetabular fractures.

Acetabular fractures were classified according to the Judet and Letournel system. The most common patterns were posterior wall fractures in four patients (18.2%), transverse fractures in four patients (18.2%), and both column fractures in four patients (18.2%). T-type fractures were seen in three patients (13.6%), anterior column fractures in three patients (13.6%), and posterior column fractures in two patients (9.1%). Less common types included anterior column with posterior hemitransverse fractures in one patient (4.5%) and unclassified variants in one patient (4.5%). The distribution of acetabular fractures is shown in Table 3.

**Table 3:** Judet and Letournel classification of acetabular fractures among the study participants T-type: transverse fracture + vertical split Judet and Letournel classification of acetabular fractures [4]

Fracture Type	Number of Cases (%)
Anterior column	3 (13.6%)
Posterior wall	4 (18.2%)
Posterior column	2 (9.1%)
Transverse	4 (18.2%)
T-type	3 (13.6%)
Anterior column with posterior hemitransverse	1 (4.5%)
Both column	4 (18.2%)
Unclassified variants	1 (4.5%)

Pelvic fractures were classified using the Tile system. The majority were Type A injuries, comprising Type A2 in 13 patients (52.0%) and Type A1 in four patients (16.0%). Unstable fracture patterns were also observed, including Type C3 in four patients (16.0%), Type B2 in two patients (8.0%), and Type C1 in two patients (8.0%). The distribution of pelvic fractures according to the Tile classification is presented in Table 4.

**Table 4:** Tile classification of pelvic fractures among the study participants Tiles classification of pelvic fractures [5]

Fracture Type	Number of Cases (%)
Tile type A1	4 (16.0%)
Tile type A2	13 (52.0%)
Tile type B2	2 (8.0%)
Tile type C1	2 (8.0%)
Tile type C3	4 (16.0%)

When assessed by the Young and Burgess classification, lateral compression injuries were the most frequent. Lateral compression type 1 was found in seven patients (28.0%), lateral compression type 2 in five patients (20.0%), and isolated rami fractures in four patients (16.0%). Vertical shear injuries were noted in two patients (8.0%), while anteroposterior compression injuries were observed in three patients (12.0%), comprising APC type 2 in two patients (8.0%) and APC type 3 in one patient (4.0%). Combined mechanism injuries accounted for four patients (16.0%). The Young and Burgess classification of pelvic fractures is detailed in Table 5.

**Table 5:** Young and Burgess classification of pelvic fractures among the study participants Young and Burgess classification of pelvic fractures [6]

Fracture Type	Number of Cases (%)
Lateral compression anterior injury (Rami fractures)	4 (16.0)
Lateral compression type 1	7 (28.0)
Lateral compression type 2	5 (20.0)
Anteroposterior compression type 2	2 (8.0)
Anteroposterior compression type 3	1 (4.0)
Vertical shear type	2 (8.0)
Combined mechanism	4 (16.0)

Out of the 47 patients, 41 (87.2%) sustained associated injuries, which included limb fractures, head injuries, chest trauma, spinal involvement, abdominal injuries, and genitourinary trauma, as detailed in Table 6. 

**Table 6:** Associated injuries among the study participants

Associated Injury	Number of Cases (%)
Limb injury	14 (34.1%)
Head injury	8 (19.5%)
Chest injury	1 (2.4%)
Abdominal injury	2 (4.9%)
Vertebral injury	5 (12.2%)
Genitourinary injury	3 (7.3%)
Maxillofacial injury	1 (2.4%)
Sciatic nerve injury	1 (2.4%)
Morel-Lavallée injury	6 (14.6%)

Six (12.8%) patients had associated comorbidities, of which two (4.3%) had type 2 diabetes mellitus, two (4.3%) had hypertension, and two (4.3%) had both diabetes mellitus and hypertension. Among the patients, 20 (42.6%) underwent surgical treatment, while 27 (57.4%) were managed non-operatively.

The assessment of patient-reported outcomes at six months revealed encouraging results. The SF-36 component scores in the 47 patients are detailed in Table 7, with physical functioning, limitations due to physical health, and emotional well-being showing high mean values, reflecting good recovery and quality of life. The mean Short Musculoskeletal Function Assessment (SMFA) score was 7.94 (range 0-48), indicating overall better health, as lower scores denote improved function. Similarly, the Oxford Hip Score (OHS) showed excellent outcomes in 24 (51.1%) patients, good in 19 (40.4%), fair in three patients (6.4%), and poor in only one (2.1%) patient. The Majeed Pelvic Outcome Grading Scale followed a comparable trend, with excellent results in 23 (48.9%) patients, good in 20 (42.6%), fair in three patients (6.4%), and poor in one (2.1%) patient. Functional outcomes assessed using the HHS showed excellent recovery in 18 (38.3%) patients, good in 25 patients (53.2%), fair in three patients (6.4%), and poor in one patient (2.1%). The Parker-Palmer Mobility Score further confirmed satisfactory outcomes, with a mean of 8.30 (SD 1.23, range 3-9), reflecting good ambulatory capacity in the majority of cases.

**Table 7:** SF-36 component scores SF-36: Short Form 36 [7]

SF-36 Items	Mean (SD)	Median (IQR)	Range
Physical Functioning	92.28 (12.85)	95 (90 - 100)	25 – 100
Limitation due to physical Health	92.98 (15.49)	95 (95 - 100)	25 – 100
Limitation due to emotional Problems	89.21 (18.70)	100 (66 - 100)	33 – 100
Energy	89.79 (5.89)	90 (85 - 95)	70 – 100
Emotional well-being	94.30 (5.06)	96 (92 - 96)	72 – 100
Social functioning	96.80 (7.60)	100 (100 - 100)	62.5 – 100
Pain	88.96 (15.24)	90 (82 - 100)	5 – 100
General health	94.26 (7.59)	95 (95 - 100)	60 - 100

Radiological assessment by Matta grading was performed in 20 patients, as several patients either declined post-operative NCCT scans or imaging was compromised by metallic implant artifacts. Among the 22 patients with acetabular fractures, 10 were managed conservatively and 12 surgically, with Matta grading available in 11 cases. In the conservatively managed group, anatomical reduction was achieved in four patients (80%) and congruent reduction in one (20%) patient. In surgically treated acetabular fractures, anatomical reduction was observed in four patients (66.6%) and congruent reduction in two (33.3%) patients. Similarly, in 21 patients with pelvic injuries, 15 underwent non-operative management while six patients required surgery; Matta grading was only possible in nine cases. Of the conservatively treated patients, one patient (20%) achieved excellent reduction and four patients (80%) good reduction, whereas among the surgically managed, two patients each had excellent and good reductions.

Correlation analysis between the generic PROM (SF-36) and the disease-specific HHS demonstrated significant associations for most domains, including physical health, emotional problems, energy, emotional well-being, social functioning, and pain, with p-values < 0.001 (Table 8). The only exception was physical functioning, which did not show a statistically significant correlation (p = 0.679). It was also noted that patients with associated injuries tended to have lower PROM scores, suggesting that concomitant trauma adversely impacted recovery, although subgroup analysis was not performed, as the study primarily aimed to assess PROMs in the overall patient cohort.

**Table 8:** Correlation between Short Form-36 component scores and the Harris Hip Score Harris Hip Score [8]

Harris Hip Score	Test Statistic (Spearman’s Rank Correlation Coefficient)	p-value
Physical Functioning	-0.062	0.679
Limitation due to Physical Health	0.619	0.001
Limitation due to Emotional Problems	0.605	0.001
Energy	0.627	0.001
Emotional Well-Being	0.747	0.001
Social Functioning	0.559	0.001
Pain	0.407	0.001

## Discussion

Fractures of the pelvis and acetabulum pose significant clinical challenges due to the integral role of the acetabulum in weight-bearing and the transference of lower limb forces to the axial skeleton through the sacroiliac joint. These injuries become even more relevant in developing countries, where high-velocity road traffic accidents and falls from height contribute to their increased incidence.

The complexity of pelvi-acetabular fractures is further compounded by the presence of extra-skeletal pelvic trauma, which can include associated lower limb fractures, genitourinary injuries, abdominal injuries, chest injuries, and head injuries. These associated injuries pose a considerable challenge when assessing the outcomes of pelvi-acetabular fractures. Since associated injuries are common and validated pelvis-specific outcome measures are lacking, evaluating outcomes in pelvi-acetabular fractures remains a challenging task.

The majority of patients in the present study belonged to the younger age groups, reflecting the high incidence of pelvi-acetabular fractures in young, active individuals.

In the current study, the PROMs revealed favorable patient outcomes. SF-36 scores assessed at the end of six months indicated better health among the patients. Similarly, SMFA scores showed lower mean values, reflecting better general health. This trend was consistent in other PROM scores. OHS and Majeed pelvic outcome grading were predominantly excellent and good, with only a few patients receiving fair or poor scores. Additionally, the HHS, a clinician-based outcome measure, also demonstrated favorable functional outcomes, with most patients achieving excellent or good scores.

Radiological assessment, utilizing the Matta grading system, was also satisfactory. Most patients with acetabular fractures achieved anatomical reduction, and some even exhibited congruent hips compared to the contralateral normal hip. This trend extended to patients with pelvic fractures as well.

Hoeksma et al. compared the responsiveness of the HHS and SF-36 in 75 patients, with a mean age of 72 years. They found that the HHS was more responsive than SF-36. In our study, the agreement between SF-36 and the HHS was found to be significant except for physical functioning. This could be because they assessed responsiveness in patients who already had osteoarthritis of the hip. In our study mean age group was 35.5 years as compared to their mean age of 72 years [10].

Kerschbaum et al. assessed the SF-36 in 105 post-operative pelvic fracture patients with a mean age of 56 years and a follow-up period of 60.5 months. The mean physical function component was 37.9. However, in the current study population, which has a mean age of 35.5 years, we found a mean of 92.28. This difference may be due to non-alignment of the mean age group and long-term follow-up in their study [11].

Pascarella et al. used SF-36 as an outcome measure in post-operative acetabular patients. The study found that the physical component of SF-36 mean was 52.9. However, in the current study, the mean was higher, which may be due to the younger mean age group [12].

Frietman et al. reported a good or better functional outcome using a modified HHS (the physical examination component of HHS is removed) in 122 of 164 (74%) post-operative patients of acetabular fractures at a mean follow-up of six years while the current study shows good or better functional outcome using the HHS in 43 out of 47 cases of pelviacetabular fractures at a mean follow up of six months [13]. 

Guo et al. applied Matta grading and Majeed Score in post-operative assessment of 18 patients with pelvi-acetabular fractures. All 18 patients had satisfactory reduction according to Matta score. Thirteen patients (72.2%) had an excellent Majeed score and five patients (17.8%) had a good Majeed score [14].

Banierink et al. conducted a systematic review of literature, including 3049 patients with pelvic ring injuries. Most studies were heterogeneous, with small cohorts of patients; the Majeed Pelvic score ranged from 75 to 95, and the SF-36 Physical functioning component ranged from 53 to 69 [15].

Hernefalk et al. found that in patients with pelvic ring fractures, male patients reported better outcomes than female patients in PROMs assessed with SF-36. However, the current study shows no significance of sex in the SF-36 outcome [16].

While this study provides valuable insights into patient-reported outcomes following pelvi-acetabular fractures, several limitations should be acknowledged. The short duration of the follow-up period may not capture long-term outcomes fully. Additionally, the study did not account for confounding factors such as associated lower limb skeletal injuries, chest, abdominal, or genitourinary injuries. Furthermore, the patients were not categorized based on their mode of treatment (non-operative or surgical). Matta grading was done using conventional radiographs, with its limitations, as some patients refused post-operative NCCT pelvic scans, and in many cases, grading could not be done on CT due to artifacts from metallic implants.

Given the limited existing literature on PROMs associated with pelvi-acetabular fractures, this study further contributes to our understanding of healthcare outcomes. The strength of this study lies in the fact that it is a prospective study and it was conducted in a setup where there is a high volume of patients with these injuries due to road traffic accidents and falls from height, and the utilization of well-established treatment protocols. However, it calls for further research with larger sample sizes and longer follow-up periods in homogeneous patient groups to provide a more comprehensive picture.

## Conclusions

This study evaluates PROMs following pelvi-acetabular fractures in patients admitted to a tertiary care hospital. It examines both generic PROMs, such as the SF-36, and disease-specific PROMs, like the HHS, to assess outcomes at a minimum follow-up of six months. The findings reveal favourable patient-reported and functional outcomes at the six-month follow-up. There is also significant agreement between the generic and disease-specific PROMs. The results indicate that patients with pelvi-acetabular trauma experience overall better health and satisfaction following treatment at the end of six months. 

## Appendices

 Proforma for patient data collection

https://drive.google.com/file/d/11SHgx1yy6qYvn7yVHdu4296rxUeScu8u/view?usp=drivesdk
